# Biologic and regenerative strategies for osteoarthritis: advances in MSC, exosome, and PRP based therapies

**DOI:** 10.3389/fphar.2026.1897284

**Published:** 2026-08-20

**Authors:** Haiyuan Yue, Ahmad Alhaskawi, Sohaib Hasan Abdullah Ezzi, Jianwei Zhou

**Affiliations:** 1 Department of Orthopaedics, The Second Affiliated Hospital of Lanzhou University, Clinical Medical School, Lanzhou, Gansu, China; 2 Orthopaedic Clinical Research Center of Gansu Province, Lanzhou, Gansu, China; 3 Intelligent Orthopaedic Industry Technology Center of Gansu Province, Lanzhou, Gansu, China; 4 Department of Orthopedics, The First Affiliated Hospital of Zhejiang University School of Medicine, Hangzhou, China; 5 Department of Orthopedics, The Fourth Affiliated Hospital of Zhejiang University School of Medicine, Yiwu, China

**Keywords:** articular cartilage, bone marrow aspirate concentrate, exosomes, mesenchymal stromal cells, osteoarthritis, platelet-rich plasma, regenerative medicine

## Abstract

Osteoarthritis (OA) is a prevalent degenerative joint disorder characterized by progressive cartilage destruction, chronic inflammation, pain, and functional impairment. Conventional therapies primarily focus on symptom management and are unable to restore damaged articular cartilage or effectively modify disease progression. Consequently, increasing attention has been directed toward biologic and regenerative approaches that target the underlying mechanisms of cartilage degeneration and joint dysfunction. This review provides a comprehensive overview of emerging regenerative strategies for articular cartilage repair, including gene- and noncoding RNA-based therapies, platelet-rich plasma (PRP), bone marrow aspirate concentrate (BMAC), mesenchymal stromal cells (MSCs), MSC-derived exosomes, and cell-based interventions. Current evidence indicates that these therapies exert their effects through modulation of inflammatory pathways, enhancement of extracellular matrix synthesis, promotion of chondrocyte survival, and regulation of tissue repair processes. Among them, PRP and BMAC offer minimally invasive approaches with favorable safety profiles, whereas MSCs and exosome-based therapies demonstrate substantial regenerative and immunomodulatory potential. Gene and epigenetic therapies further provide opportunities to target disease-associated molecular pathways and improve cartilage homeostasis. Despite encouraging preclinical and clinical outcomes, significant challenges remain, including heterogeneity in biologic preparations, variability in treatment protocols, limited long-term clinical evidence, and the absence of standardized outcome measures. Future research should focus on mechanistic validation, protocol standardization, optimization of delivery strategies, and large-scale randomized clinical trials to establish the long-term safety and disease-modifying efficacy of these regenerative therapies. Collectively, biologic and regenerative interventions represent promising avenues for advancing cartilage repair and improving clinical outcomes in patients with osteoarthritis.

## Introduction

Osteoarthritis (OA) is a condition that impacts millions of individuals globally (Felson et al., 2000; Bijlsma et al., 2011; Chevalier et al., 2013). It results in pain associated with movement, progressive functional impairment, and a diminished quality of life (Bijlsma et al., 2011; Chevalier et al., 2013; Corti and Rigon, 2003; Pottie et al., 2006). OA is now recognized as a complex, multifactorial condition that impacts the entire joint, encompassing the bone, cartilage, ligaments, and muscles (Wieland et al., 2005; Hunter and Bierma-Zeinstra, 2019; Tang et al., 2025). At present, the most effective treatments for OA focus on prophylactic or preventive strategies aimed at inhibiting the onset or slowing the progression of the degenerative process, a concept known as chondroprotection (Murray et al., 2016; Chahla and Mandelbaum, 2018). According to the Osteoarthritis Research Society International (OARSI) and the American Academy of Orthopaedic Surgeons (AAOS), the primary approaches to managing OA include physical interventions, pharmacological therapies, and surgical procedures (Zhang et al., 2008; Jevsevar et al., 2013). Pharmaceutical therapy remains the most frequently utilized treatment for OA, primarily focusing on pain relief and inflammation reduction. Traditional OA medications are limited in their ability to manage symptoms and do not address the underlying joint damage. Furthermore, these conventional drugs are often associated with a high incidence of adverse effects. Ongoing research is exploring new OA therapies, particularly biologic agents, which aim to offer greater efficacy with fewer side effects. Additionally, regenerative therapy presents a promising approach by potentially repairing and regenerating damaged or degenerated tissues, with the goal of restoring the original structure and function of the joint (Mason and Dunnill, 2008). In recent years, regenerative and biologic therapies have received increasing scientific and clinical attention for their potential to modulate joint homeostasis, enhance intrinsic repair mechanisms, and attenuate the progression of structural degeneration (Lanza et al., 2020; Zhang et al., 2016). The number and degranulation status of mast cells (MCs) were positively correlated with the synovitis score and cartilage injury (Hao et al., 2024; de Lange-Brokaar et al., 2016). MCs interact with inflammatory cells, leading to changes in the synovial fluid microenvironment and facilitate the progression of synovial inflammation (Hao et al., 2024; Kulkarni et al., 2022). Notably, MCs-produced IL-1β, TNF-α, and IL-6 can activate macrophages, causing them to produce large quantities of proinflammatory cytokines (TNF-α, IL-1, IL-12, IL-18, and IFN-γ), chemokines, and MMPs in the synovitis environment, ultimately leading to osteoclast formation, erosion, and progressive joint destruction (Hao et al., 2024; Wang et al., 2014; C et al., 2023). Therefore, the present review critically synthesizes current advances in platelet-rich plasma (PRP), bone marrow aspirate concentrate (BMAC), mesenchymal stromal cells (MSCs), extracellular vesicles/exosomes, and gene-based interventions. By integrating mechanistic insights with available clinical data, we aim to provide a comprehensive evaluation of their biological rationale, therapeutic potential, and safety profiles within the context of knee osteoarthritis.

## Search strategy

**FIGURE 1:**
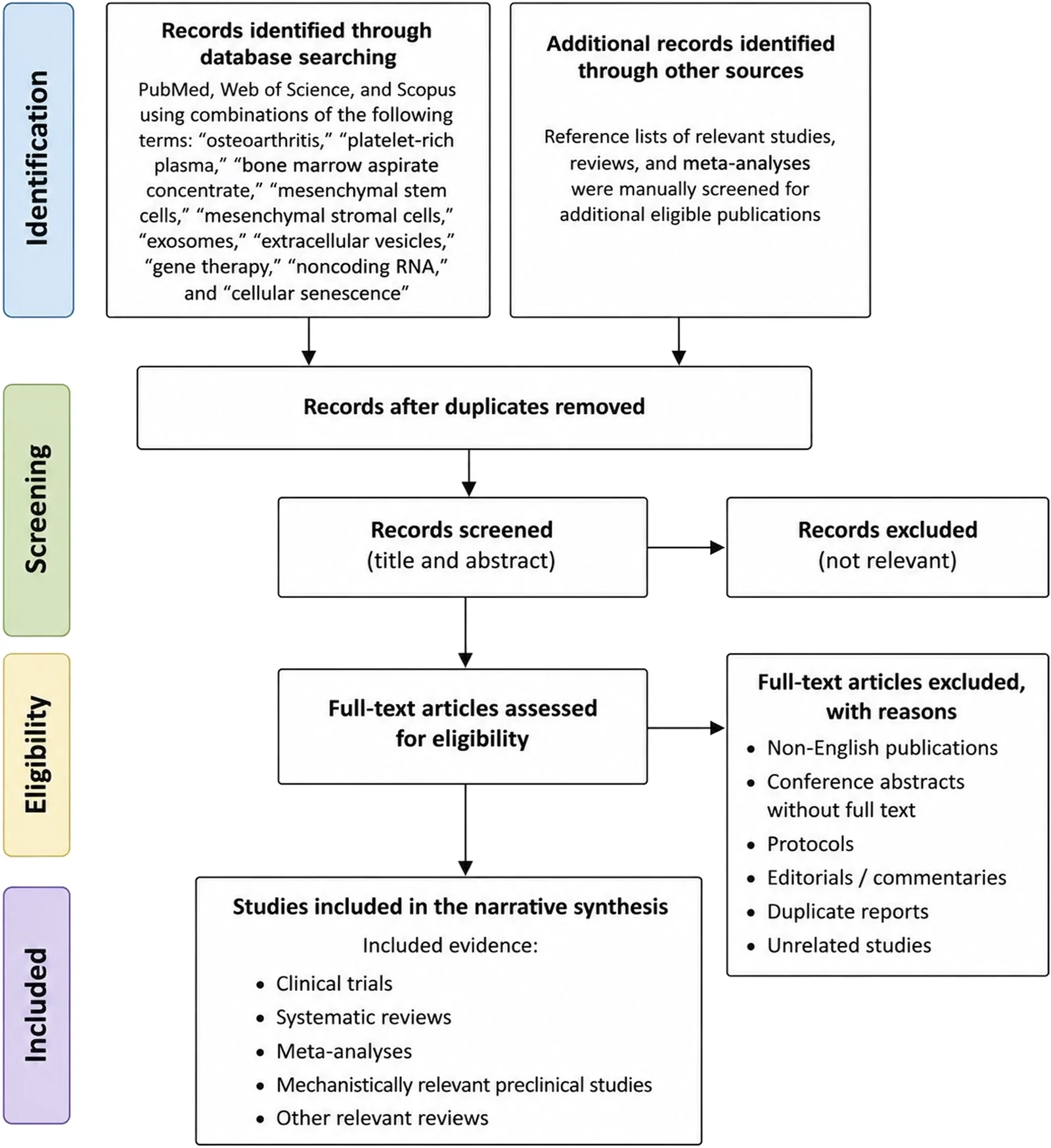
PRISMA-style flow diagram summarizing the identification, screening, eligibility assessment, and final inclusion of studies in the narrative synthesis.

This narrative review was supported by a structured search of PubMed, Web of Science, and Scopus using combinations of the terms “osteoarthritis,” “platelet-rich plasma,” “bone marrow aspirate concentrate,” “mesenchymal stem cells,” “mesenchymal stromal cells,” “exosomes,” “extracellular vesicles,” “gene therapy,” “noncoding RNA,” and “cellular senescence,“. Clinical trials, systematic reviews, meta-analyses, and mechanistically relevant preclinical studies were included, whereas non-English publications, conference abstracts without full text, protocols, editorials, duplicate reports, and unrelated studies were excluded. Duplicates were removed before title, abstract, and full-text screening, and reference lists were additionally examined for relevant studies (Figure 1). As this was a narrative review, independent dual-reviewer screening and formal risk-of-bias assessment were not performed.

## Cellular and molecular mechanisms of osteoarthritis

Before discussing specific regenerative and biologic strategies, it is essential to outline the cellular and molecular mechanisms that underlie cartilage degeneration in OA, as these pathways represent the principal targets of the therapeutic agents discussed below. Pro-inflammatory cytokines, particularly IL-1β, TNF-α, and IL-6, are key mediators that stimulate chondrocytes to upregulate degradative enzymes such as matrix metalloproteinases (MMP-1, MMP-3, MMP-13) and aggrecanases (ADAMTS-4 and ADAMTS-5); these enzymes target and cleave aggrecan and collagen fibrils, leading to loss of tensile strength, increased water content, and impaired load-bearing capacity of the matrix (Qiu et al., 2026; Yu et al., 2020; Charlier et al., 2019). During OA progression, chondrocytes cluster, become hypertrophic, or dedifferentiate into fibroblasts and become apoptotic; chondrocytes with a hypertrophic phenotype produce type X collagen and higher levels of cartilage-degrading enzymes than normal chondrocytes (Sengprasert et al., 2023). In parallel, cellular senescence contributes substantially to disease progression: inflammation of joint tissues induces the hallmarks of senescence in resident cells, which further facilitates the senescence-associated secretory phenotype (SASP), namely, the secretion of chemokines, cytokines, and growth factors, leading to recruitment of inflammatory cells, reactive oxygen species generation, extracellular matrix degradation, and subchondral osteosclerosis (Han et al., 2024). Senescent chondrocytes further exert a pro-senescence effect on surrounding normal chondrocytes via SASP, while senescent synovial cells act on chondrocytes through cytokines such as TNF-α, VEGF, and IL-6, propagating cartilage degeneration through intercellular communication (Wu et al., 2022a). Subchondral bone remodeling represents an additional contributory mechanism, whereby subchondral bone cells promote osteoclast activity through RANKL; overactivated osteoclasts enhance bone resorption and secrete factors that promote subchondral bone remodeling and further aggravate OA pathological progression [Bone Research, (Wu et al., 2022a). Importantly, the detrimental effects of senescence are primarily attributable to impaired regenerative capacity of stem cells and the pro-inflammatory environment created by accumulated senescent cells. However, removal of senescent cells and suppression of SASP factors have shown promise in mouse models; clinical trials have yet to demonstrate significant efficacy, underscoring the gap between mechanistic understanding and successful clinical translation that the present review aims to address (Xiong et al., 2025).

## Recent progress in biological interventions

### DNA- or gene-based therapy

DNA, a double-stranded, elongated polymer composed of four deoxynucleotides, serves as the foundation for genetic material, with segments encoding information termed genes (Minchin and Lodge, 2019). Numerous genes have been implicated in the etiology and progression of OA through diverse mechanisms, including heightened susceptibility, accelerated degradation of the cartilaginous matrix, inhibition of cartilage repair, upregulation of inflammatory mediators, and facilitation of fibroblast transformation. Susceptibility to OA is associated with genes such as ASPN (Wang et al., 2018), ADIPOQ (Shang et al., 2019), AKNA (Zhao et al., 2020), DPEP1 (Zhang et al., 2021), rs1065080 (Lu et al., 2019), TLR7, CRIP1 (Wang et al., 2020a), ZNF688, TOP1, EIF1AY, RAB2A, RTP4, AGT, ZNF281, UIMC1 (Wang et al., 2020b), and PRKACB (Zhao, 2021). Cartilage degradation is driven by genes, including ADAMTS5 (Jiang et al., 2021), ADAM12, JUN, PTGS2, MMP1, MMP3, MMP13 (Zhou et al., 2019), and rs2830585 (Zhou et al., 2019). Genes such as BMP3 (He et al., 2018), rs1799750 (Geng et al., 2018), and CHI3L1 (Song et al., 2021) impede cartilage repair, while inflammatory cytokine expression in chondrocytes is regulated by genes like renin, ACE, Ang II, AT1R, AT2R, ATF3 (Wu et al., 2019), PTGS2 (Wang et al., 2020b), CCL20, CHI3L1, LIF, CXCL8, and CXCL12 (Lin et al., 2018). Additionally, fibroblast transformation involves COL6A3/ACTG1 and FN1 (Lin et al., 2018). Although catabolic genes are well-documented, anabolic genes promoting chondrocyte proliferation, differentiation, or encoding critical collagen-anchoring molecules such as GDF5 (Sun et al., 2021), Gas7 (Zhong et al., 2020), PRELP (Li et al., 2019), TGF-β, SOX9, and COL9A1; remain scarce (Durand et al., 2020). Preclinical studies demonstrate that both *ex-vivo* and *in-vivo* gene delivery approaches can successfully modify joint tissues, with adeno-associated viral (AAV) vectors showing particular promise for OA therapy. Direct intra-articular AAV administration has emerged as a practical and effective strategy for enhancing cartilage repair and regulating disease-related genetic pathways due to the vector’s favorable safety profile, high transduction efficiency, and low immunogenicity (Evans et al., 2018). These vectors offer the potential for prolonged transgene expression, effectively transducing synovial lining cells and chondrocytes across the depth of the AC (Watson Levings et al., 2018). Beyond preclinical vector development, gene therapy has progressed furthest toward clinical translation among disease-modifying OA candidates: TissueGene-C, an allogeneic chondrocyte-based gene therapy engineered to overexpress TGF-β1, has advanced through phase 2 and phase 3 clinical trials, with reported improvements in pain and function in patients with knee OA (Morici and Nikolic, 2026). Epigenetic regulation also plays a significant role in OA pathogenesis, with DNA methylation emerging as a key mechanism that alters the expression of genes essential for cartilage integrity. Notably, hypermethylation of COL9A1 reduces its expression, weakening collagen anchoring within the extracellular matrix and contributing to structural deterioration and disease progression (Miranda-Duarte, 2018). SOX9, a key transcription factor required for cartilage development and maintenance, undergoes promoter hypermethylation in OA chondrocytes, reducing its binding affinity to target genes and thereby diminishing its expression and downstream chondrogenic activity (He et al., 2020). DNA methyltransferases represent potential therapeutic targets for future OA interventions.

### Noncoding RNA-based therapy

**FIGURE 2:**
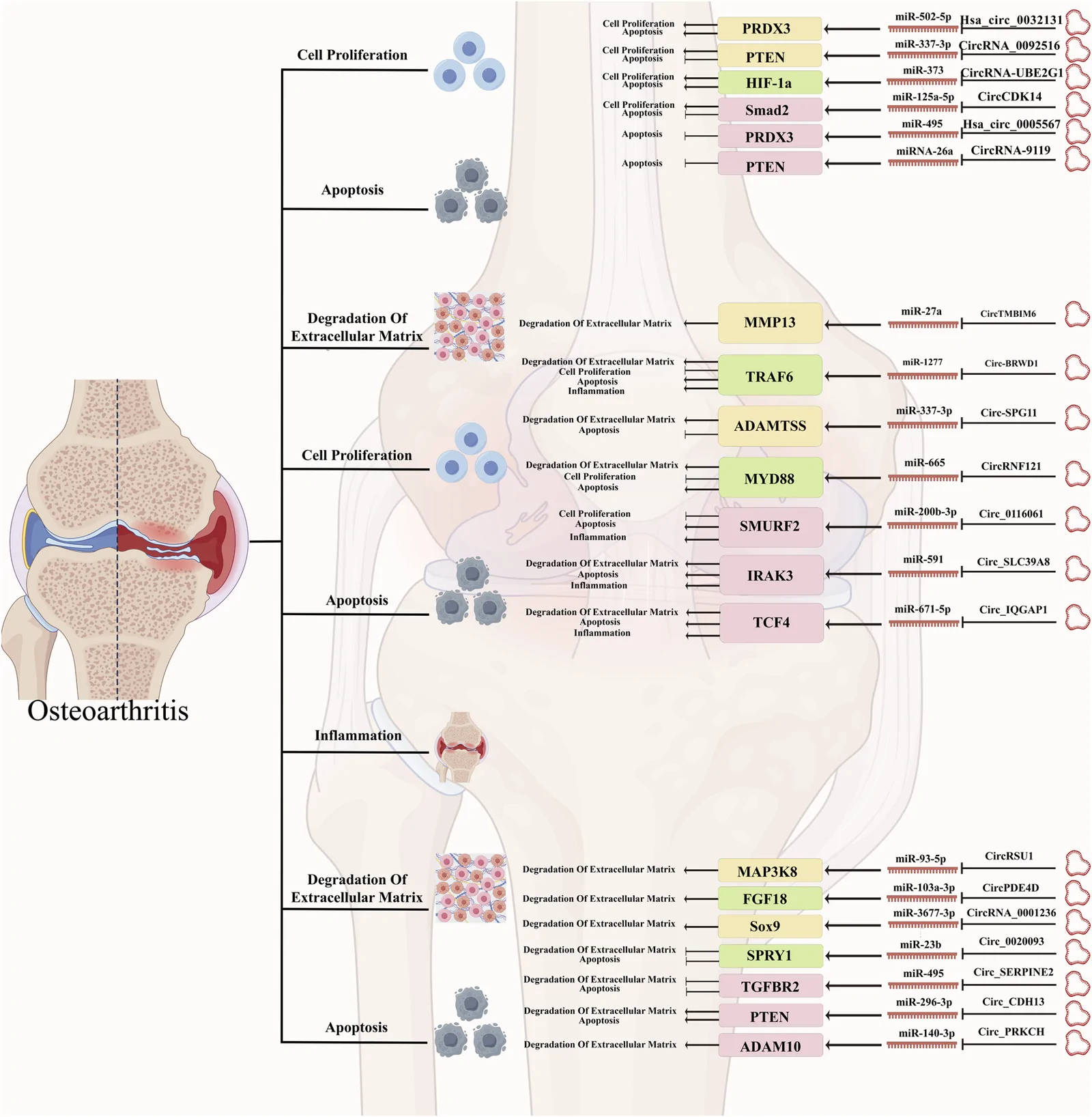
Interactions between circRNAs, miRNAs, and target genes driving osteoarthritis progression.

Epigenetic mechanisms, particularly those mediated by noncoding RNAs (ncRNAs), have gained substantial attention in OA research due to their therapeutic potential. ncRNAs, including miRNAs, lncRNAs, and circRNAs constitute the majority of the human transcriptome and have a pivotal roles in regulating gene expression pathways that drive cartilage degeneration and repair in OA (Duan et al., 2020). These ncRNAs serve as diagnostic and prognostic markers and influence key gene expression, offering translational promise for OA management. Elucidating ncRNA roles could enhance understanding of OA etiology and inform therapeutic target development, though their association with oncogenesis necessitates rigorous preclinical safety assessments (He et al., 2021; Xie et al., 2020a). Among ncRNAs, miRNAs short, approximately 22-nucleotide sequences have gained prominence for their roles in RNA silencing and posttranscriptional gene regulation. Multiple studies highlight miRNAs’ significance in maintaining bone and cartilage homeostasis by modulating signaling pathways linked to ECM degradation, chondrocyte apoptosis or hypotrophy, and synovial inflammation (Shen et al., 2019). LncRNAs, exceeding 200 nucleotides in length, regulate mRNA translation and degradation or act as miRNA sponges (Zhang et al., 2021). Critical to cartilage development, lncRNAs may counter OA by competitively binding miRNAs, thereby reducing miRNA interactions with downstream genes and enhancing their transcription and expression (Wu et al., 2019). CircRNAs, covalently closed molecules containing exon sequences spliced at canonical sites, function as miRNA sponges or competing endogenous RNAs, inhibiting miRNA activity (Tam et al., 2019). Emerging as novel contributors to OA, circRNAs influence apoptosis, chondrocyte proliferation, ECM degradation, and inflammation (Yang et al., 2021a). To date, ncRNA-based therapeutics for OA remain at the preclinical stage, and no miRNA-, lncRNA-, or circRNA-targeted agent has yet advanced to human clinical trials for this indication. Several specific circRNA-miRNA-target gene axes illustrate this regulatory network (Figure 1). CircTMBIM6 is overexpressed in OA cartilage and promotes extracellular matrix degradation by sponging miR-27a, thereby relieving its suppression of MMP13 and increasing collagenolytic activity (Bai et al., 2020a). In a converse, chondroprotective example, circRNA-9119 sequesters miR-26a, preventing its inhibition of PTEN and thereby protecting IL-1β-stimulated chondrocytes from apoptosis (Chen et al., 2020a). A third axis, circRNA-UBE2G1/miR-373/HIF-1a, has been implicated in inflammatory chondrocyte injury: circRNA-UBE2G1 sponges miR-373 to upregulate HIF-1a, increasing pro-inflammatory cytokine production (IL-1β, IL-6, TNF-α) and chondrocyte apoptosis under lipopolysaccharide stimulation (Chen et al., 2020b). These examples illustrate how individual circRNA-miRNA axes can exert either catabolic/pro-apoptotic or protective effects depending on their downstream target, underscoring the regulatory complexity depicted in Figure 2. To date, ncRNA-based therapeutics for OA remain at the preclinical stage, and no miRNA-, lncRNA-, or circRNA-targeted agent has yet advanced to human clinical trials for this indication.

### Protein-based therapy

Platelet-rich plasma (PRP), an autologous preparation enriched with platelets exceeding baseline blood concentrations, dominates clinical protein-based interventions (Szwedowski et al., 2021). PRP’s therapeutic potential stems from its capacity to deliver supraphysiological growth factor levels, fostering tissue repair. Activation precedes its use, and intra-articular PRP injections offer a viable alternative to analgesics for KOA (Gentile et al., 2020; Rajan et al., 2020). In addition, PRP enhances chondrocyte survival and autophagy through FOXO1, FOXO3, and HIF-1 modulation, with leukocyte-poor PRP showing superior chondroprotective effects compared with leukocyte-rich formulations, and further synergistic benefit achieved when combined with hyaluronic acid (Zhao et al., 2020), though inconsistent preparation methods and components limit conclusive efficacy (Szwedowski et al., 2021). Beyond PRP, investigated proteins include nerve growth factor antibodies and antagonists (e.g., fasinumab (Dakin et al., 2021), tanezumab (Berenbaum et al., 2020)), fibroblast growth factor (FGF) (Xie et al., 2020b), insulin-like growth factor-binding proteins (IGFBP) (Tanaka et al., 2021), growth and differentiation factor 5 (Kania et al., 2020), Wnt16 (Tong et al., 2019), low-density lipoprotein receptor-related protein 5, and neuropeptide Y (NPY) (Kang et al., 2020). Clinical trials demonstrate varied effects: fasinumab and tanezumab alleviate pain, though tanezumab risks rapid OA progression; FGF, GDF5, NPY, Wnt16, sprifermin (Eckstein et al., 2020), and teriparatide (Apostu et al., 2019) support cartilage repair; IGFBP aids matrix synthesis; and low-density lipoprotein receptor-related protein-sclerostin binding curbs normal chondrocyte degradation, with unclear OA effects (Grässel and Muschter, 2020). Histone modifications, mediated by epigenetic regulators like DOT1L, EZH2, KDM4B, KDM6A, KDM6B, and LSD1, influence OA onset and progression via methyltransferase and demethylase activities targeting genes (e.g., Nfat1, Runx2, Sox9) or interacting with OA-related pathways. These modifications serve as epigenetic signatures for gene activation or repression, positioning methyltransferases and demethylases as prospective OA management targets (Sacks et al., 2018). Although PRP is generally considered a safe therapeutic option, its safety profile requires careful evaluation, particularly regarding potential adverse events such as infection, bleeding, and allergic reactions following administration in KOA patients (Rai et al., 2021). Long-term safety evaluation of PRP is essential to confirm the durability of its therapeutic effects and to identify delayed adverse events, which may be influenced by patient-specific factors such as comorbidities and medications. Ensuring ethical clinical use requires standardized safety protocols, transparent patient counseling, and continuous monitoring and reporting of complications to maintain PRP as a reliable treatment for knee osteoarthritis (Rai et al., 2021).

### Bone marrow aspirate concentrate (BMAC)

BMAC has emerged as a promising regenerative therapy for KOA, gaining significant attention due to its unique mechanism, as unlike other treatments, BMAC harnesses the regenerative potential in the patient’s BM, specifically through MSCs and growth factors (Kim et al., 2020). BMAC delivers a concentrated mixture of regenerative cells and bioactive molecules that directly targets OA degeneration, with MSCs supporting chondrogenic repair and growth factors promoting tissue healing offering a bone marrow-derived, disease-modifying alternative to symptomatic therapies (Szwedowski et al., 2021). A clearer understanding of these underlying mechanisms is essential for refining treatment protocols, improving patient selection, and ensuring safety, positioning BMAC as a potentially transformative strategy in the future management of KOA (Fayed et al., 2024). BMAC’s regenerative potential derives from its MSCs, which can differentiate into chondrocytes to support cartilage repair, alongside a rich mixture of growth factors and cytokines that modulate joint inflammation and create a pro-healing microenvironment; together, these components address key pathological features of KOA, including cartilage degradation, inflammation, and impaired joint function (Costa et al., 2022). Understanding how MSC differentiation and the coordinated actions of growth factors and cytokines drive cartilage regeneration and modulate inflammation is essential for optimizing BMAC therapy and improving regenerative outcomes in knee osteoarthritis (Fan et al., 2020). Although BMAC is generally considered safe, its use requires careful evaluation of procedural risks, including complications from bone marrow aspiration, product concentration, and intra-articular injection, as well as patient-specific factors that may influence adverse events (Muthu et al., 2023). Long-term safety monitoring is essential to detect delayed complications and to establish the durability and ethical clinical application of BMAC in KOA, emphasizing strict adherence to standardized protocols and systematic reporting to ensure it remains a reliable regenerative therapy (Yamaguchi et al., 2019).

### Combining PRP and BMAC

The combination of PRP and BMAC provides a synergistic regenerative strategy for KOA by uniting PRP’s growth-factor-rich signaling environment (including PDGF, TGF-β, and IGF) with the chondrogenic potential of MSCs in BMAC, creating a multidimensional platform that enhances tissue repair and may slow disease progression (Xu et al., 2020). This combined therapy using BMAC and PRP has demonstrated a favorable safety profile and may exert synergistic regenerative effects. Stem cells obtained from BMAC, in conjunction with the bioactive growth factors and cytokines present in PRP, could contribute to improved tissue healing and regenerative processes (Shehadi et al., 2021).

Biomaterials are often combined with PRP and BMAC to improve the delivery, retention, and activity of bioactive molecules while enhancing cell interactions. Since musculoskeletal tissues differ in structure and function, selecting an appropriate biomaterial is essential for maximizing therapeutic outcomes. In addition, the physical and biochemical properties of the biomaterial must support both the local tissue environment and the regenerative potential of PRP and BMAC (Yamaguchi et al., 2019; Lee et al., 2014). Understanding how PRP’s growth factors interact with the MSCs in BMAC is essential for clinicians to refine treatment strategies and develop more targeted, personalized approaches that maximize therapeutic benefits for patients with (Balusani et al., 2024). Both PRP and MSC secretomes downregulate TNF-α, IL-1β, and MMP-13, curbing the inflammatory-catabolic loop seen in OA (Yuan et al., 2013; Filardo et al., 2015; Caplan and Correa, 2011; van Buul et al., 2012). This combined PRP-BMAC approach targets both symptoms and underlying pathology in knee osteoarthritis by promoting tissue repair, modulating inflammation, and potentially altering disease progression, paving the way for future advances in personalized regenerative medicine (Balusani et al., 2024). A combination therapy appears to be safe and may provide synergistic therapeutic benefits. The stem cell content derived from BMAC, together with the growth factors and cytokine-rich microenvironment provided by PRP, may enhance tissue repair and regeneration. This combined therapeutic approach demonstrates promising potential for promoting recovery following spinal cord injury (SCI).

### Mesenchymal stem cell-based therapy

**FIGURE 3:**
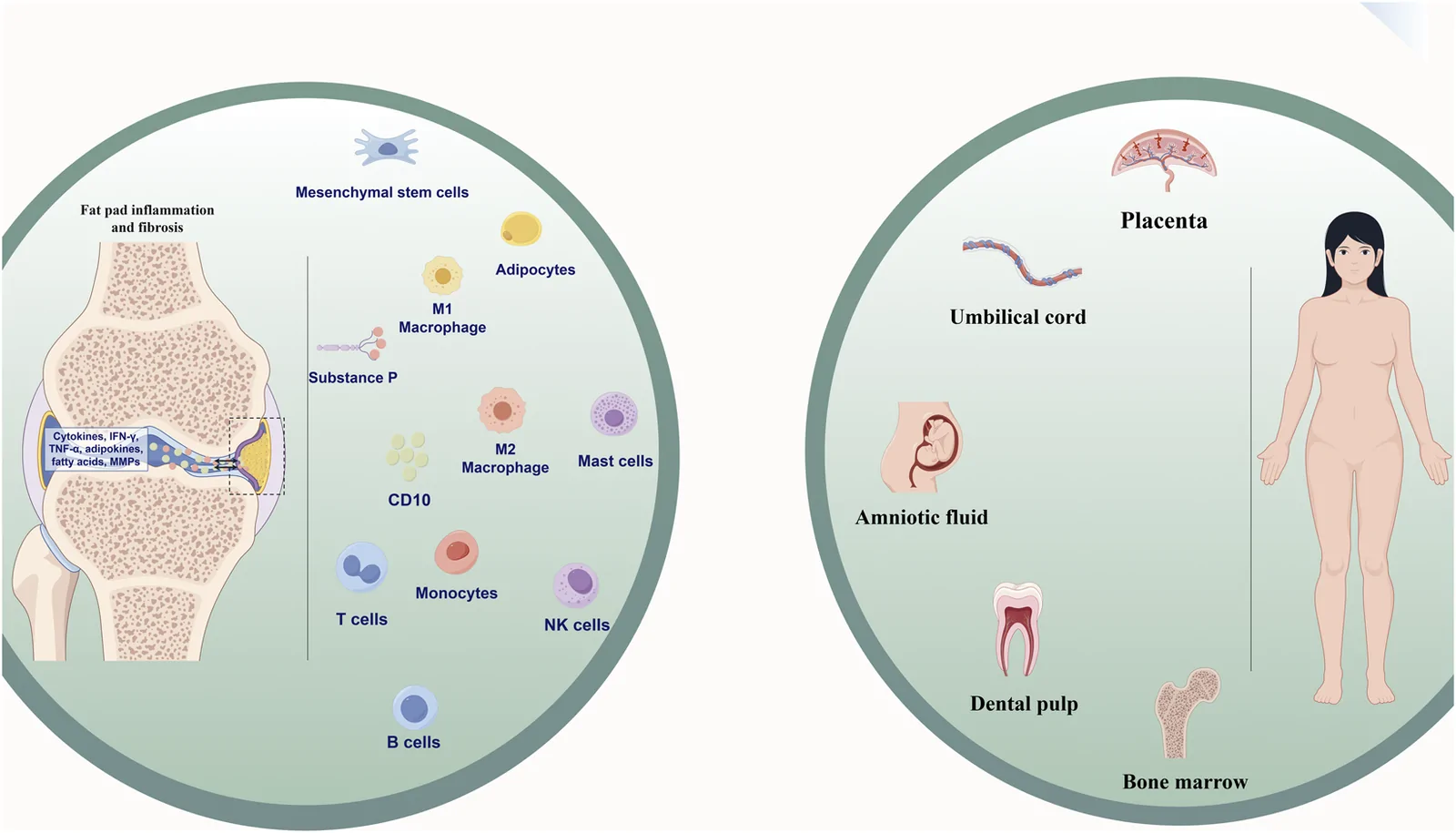
Sources and immunomodulatory interactions of mesenchymal stromal cells (MSCs) relevant to osteoarthritis. Left: infrapatellar fat pad inflammation and joint immune microenvironment involving macrophages, mast cells, T cells, B cells, NK cells, and monocytes. Right: major human tissue sources of MSCs, including bone marrow, adipose tissue, umbilical cord, placenta, amniotic fluid, and dental pulp.

MSCs can be sourced from multiple tissues, including bone marrow, adipose tissue, umbilical cord, amniotic fluid, placenta, dental pulp, and cord blood, with bone marrow and adipose tissue being the most commonly used due to their established chondrogenic potential (Figure 3). Among these sources, umbilical cord and amniotic-derived MSCs demonstrate the highest proliferative capacity and strongest immunomodulatory effects, while also secreting greater levels of growth factors compared with bone marrow-derived MSCs, making UC-MSCs particularly promising for regenerative applications in OA (Table 1) (Zhu et al., 2021).

**TABLE 1:** Characteristics of different types of stem cells (Zhu et al., 2021).

MSCs source	Advantages	Disadvantages
Bone marrow	Easy accessibility	Relatively low cell growth rate
	High multilineage differentiation	
	Relatively adequate clinical trials	
Adipose tissue	Easy accessibility	Reduced differentiation capability
	A greater number of colonies formation	Inadequacy in clinical trials
	Superior immunomodulatory capacity	
Umbilical cord blood stem cells	Longer culture times	Relatively few colonies formation
	Higher proliferation capacity	Relatively low yield
	Higher anti-inflammatory effects	Inadequacy in clinical trials

**FIGURE 4:**
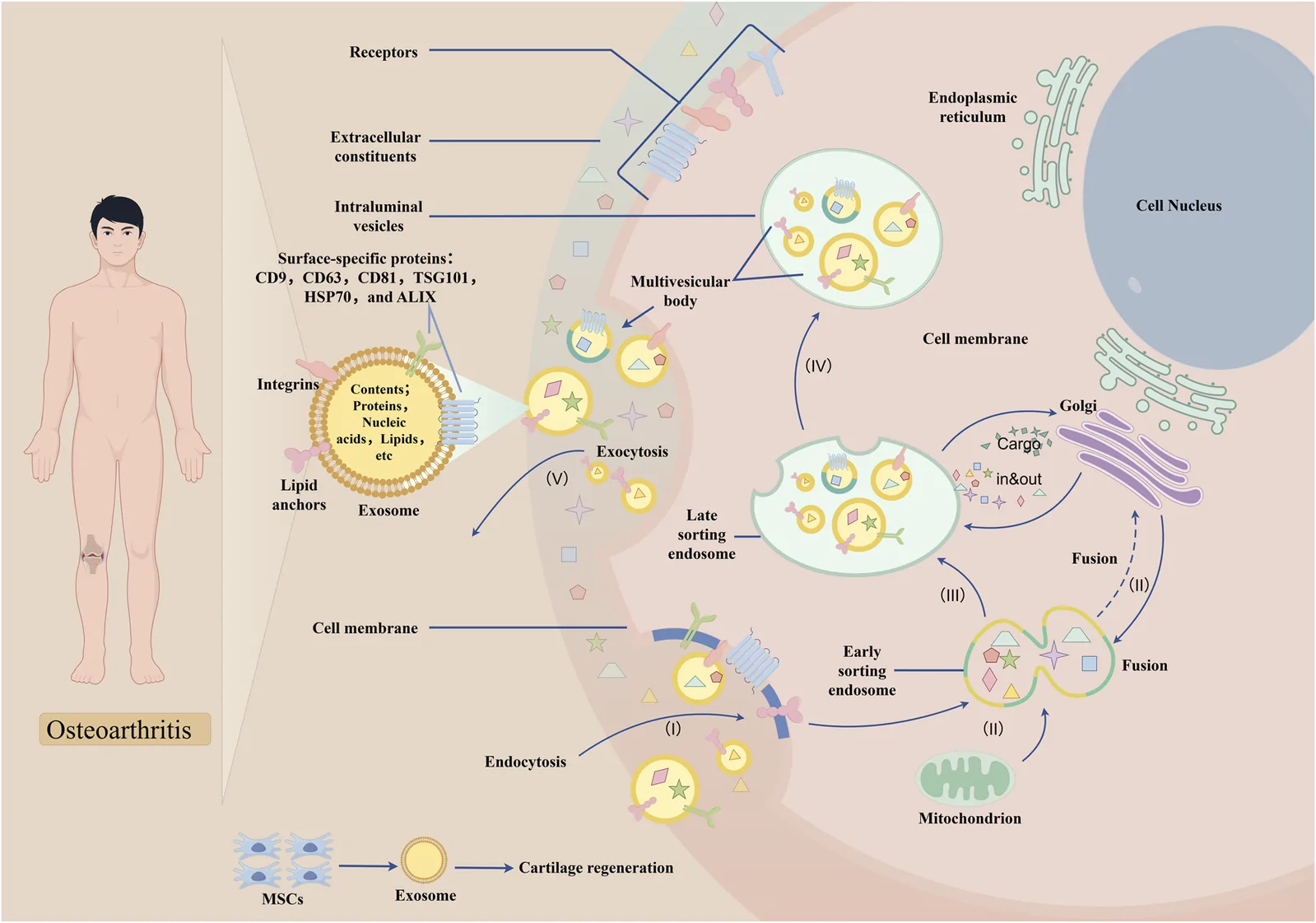
Biogenesis, maturation, secretion, and cellular uptake of exosomes, illustrating early and late endosomal formation, multivesicular body (MVB) development, plasma membrane fusion, and exosome-mediated delivery of molecular cargo. The figure also highlights MSC-derived exosome contribution to cartilage regeneration in osteoarthritis.

MSCs possess several defining biological characteristics that underpin their therapeutic potential in knee osteoarthritis. Their inherent plasticity allows them to self-renew, maintain stemness, and differentiate into various mesodermal tissues, while also migrating to sites of injury where they exert potent trophic and regenerative effects (Gherghel et al., 2023). Once within the joint microenvironment, MSCs modulate numerous proliferative, angiogenic, and reparative pathways through the secretion of key mediators such as G-CSF, SCF, M-CSF, and IL-6, while simultaneously suppressing pro-inflammatory cytokines including TNF-α (Gherghel et al., 2023; Chen et al., 2026). A major component of their therapeutic activity arises from their immunomodulatory capacity: MSCs interact with lymphocytes, B cells, dendritic cells, and natural killer cells, and they migrate to inflamed tissues to regulate cytokine networks, inhibit the maturation of antigen-presenting cells, and promote the expansion of CD4^+^CD25^+^ regulatory T cells (Luque-Campos et al., 2019). Although MSCs are capable of differentiating into multiple musculoskeletal cell types, such as myocytes, tenocytes, and ligament fibroblasts, accumulating evidence indicates that their benefits in osteoarthritis stem predominantly from indirect trophic mechanisms rather than direct cartilage regeneration (Gherghel et al., 2023). The tropic properties of MSCs, rather than their chondrogenic differentiation, likely promote cartilage repair. Wu et al. (2011) demonstrated that MSCs stimulate chondrocyte proliferation and ECM deposition by secreting water-soluble compounds. Recent studies, such as the work by Pak (2011), have shown positive results in KOA patients treated with MSCs combined with hyaluronic acid (HA), dexamethasone, and PRP, leading to improvements in pain, function, and cartilage thickness. However, determining the exact contribution of each therapy remains challenging due to the combined nature of the treatment approach. A five-year follow-up study by Davatchi et al. demonstrated that MSC treatment resulted in sustained improvements in pain, function, and physical performance in KOA patients, highlighting the long-term potential of MSC-based therapy (Davatchi et al., 2016). However, variations in MSC administration methods and dosages complicate the ability to compare clinical outcomes effectively, underscoring the need for more research to determine optimal MSC dosages and delivery methods (Wolfstadt et al., 2015). Although MSC therapy is promising, uncertainties remain because metabolic pathways can alter transplanted cell behavior, and no current method reliably controls their chondrogenic differentiation, limiting predictability in cartilage repair applications (Gherghel et al., 2023). Furthermore, various MSC sources have been explored for OA therapy, including bone marrow-derived MSCs (BM-MSCs), adipose-derived MSCs (AD-MSCs), and umbilical cord MSCs (UC-MSCs). BM-MSCs remain the most widely used and well-characterized source, exhibiting strong proliferative capacity, robust differentiation potential, and low immunogenicity. Early clinical studies, including those involving patients undergoing high tibial osteotomy, have shown hyaline-like cartilage regeneration and improved histological features, reinforcing their therapeutic promise (Volarevic et al., 2017). AD-MSCs display even greater proliferative and differentiation potential, and a Phase I dose-escalation trial in severe KOA demonstrated meaningful improvements in pain and function with a favorable safety profile, particularly at lower cell doses (Pers et al., 2016). In addition, combining AD-MSCs with microfracture procedures has been shown to enhance KOOS pain and symptom scores at 24 months, although broad functional outcomes did not significantly improve, suggesting selective benefits for tissue repair and pain reduction (Koh et al., 2016). UC-MSCs have also gained attention due to their superior proliferative and immunomodulatory characteristics, with clinical data showing significant improvements in Lysholm, WOMAC, and SF-36 scores at 6 months, accompanied by only transient post-injection pain or swelling (Saghahazrati et al., 2020). Strategies to enhance MSC function, such as low-level laser pretreatment, have been proposed; this approach stimulates endothelial cells, fibroblasts, osteoblast-like cells, and MSCs by increasing growth factor release, nitric oxide production, reactive oxygen species, ATP, and nucleic acids. However, despite these advances, limitations such as low *in vitro* yields and restricted proliferative capacity continue to pose challenges to the large-scale translation of MSC-based therapies (Abdallah et al., 2019). Increasing interest has also focused on MSC-derived exosomes, nanosized (30–200 nm) extracellular vesicles that mirror the molecular profile of their parent cells and carry diverse bioactive cargos including glycoconjugates, lipids, nucleic acids, and proteins (Pegtel and Gould, 2019). These vesicles contain conserved sets of membrane trafficking and fusion molecules such as Rab GTPases, annexins, integrins, and fibronectin. Exosome biogenesis originates from the double invagination of the plasma membrane leading to early endosome formation, which subsequently matures into multivesicular bodies (MVBs) containing intraluminal vesicles-later released as exosomes (Meldolesi, 2018; van Niel et al., 2018; Wu et al., 2022b; Jin et al., 2024). Functionally, exosomes participate in intercellular communication via membrane fusion or ligand-receptor interactions (Wu et al., 2020) and can be derived from diverse tissues including peripheral blood (Ni et al., 2020; Chang et al., 2018), synovial fluid (Gao et al., 2020), MSCs (Tofiño-Vian et al., 2018), embryonic stem cells, vascular endothelial cells, dental pulp stem cells, monocytes (Bai et al., 2020b), amniotic fluid stem cells (Beretti et al., 2018), chondrogenic progenitor cells, chondrocytes (Zheng et al., 2019), PRP (Liu et al., 2019), osteocytes (Lyu et al., 2020). Their roles vary by origin, influencing OA development within the joint microenvironment. Therapeutically, exosomes promote chondrogenic marker expression and cartilage ECM production or suppress inflammation, hypertrophy, and apoptosis, highlighting their potential (Zhou et al., 2020) (Figure 4).

### Exosome-based therapy: clinical translation and challenges

Conventional post-secretion cargo-loading methods, including electroporation, sonication, extrusion, freeze-thaw cycling, and chemical permeabilization, may exhibit limited loading efficiency, uneven cargo distribution, vesicle aggregation, membrane damage, and cargo leakage, while also complicating the removal of free therapeutic agents and unloaded vesicles (Zhu et al., 2026). Endogenous engineering provides an alternative strategy by exploiting the cellular pathways that naturally regulate protein and RNA sorting into exosomes, including ESCRT-dependent and ESCRT-independent mechanisms, the syndecan-syntenin-ALIX axis, tetraspanin-enriched membrane domains, and RNA-binding proteins. Engineering approaches based on CD63 or LAMP2B fusion proteins, RNA-recognition motifs, KFERQ-mediated sorting, and RNA-binding protein–tetraspanin systems may improve the selective enrichment of therapeutic proteins or RNAs while preserving vesicle integrity. Surface presentation of chondrocyte-affinity peptides may additionally improve the targeting of engineered vesicles to cartilage cells (Zhu et al., 2026).

Clinical translation of exosome-based therapy for OA remains in its earliest stages (Table 2). One of the first published clinical trials evaluated intra-articular human umbilical cord MSC-derived exosomes (hUC-MSC-Exos) in patients with mild to moderate knee OA. In this randomized, double-blind, ascending-dose study, 41 patients with 45 affected limbs received low- (3 × 10^11^), medium- (4 × 10^11^), or high-dose (5 × 10^11^) exosome particle injections into the joint cavity on days 0, 21, and 42, following preclinical validation in mouse OA models and human chondrocyte toxicity testing (Wang et al., 2025).

**TABLE 2:** Catabolic and anabolic roles of exosomes in osteoarthritis.

Functions	Origins	Mechanisms
Catabolic effect	Synovial fluid (Gao et al., 2020), vascular endothelial cells (Yang et al., 2021a)	Recruit inflammatory cells (Gao et al., 2020), inhibit cartilage proliferation (Gao et al., 2020), promote joint degeneration (Gao et al., 2020), or induce chondrocyte apoptosis
Anabolic effect	MSCs (Tofiño-Vian et al., 2018), embryonic stem cells, dental pulp stem cells, monocytes (), amniotic fluid stem cells (Beretti et al., 2018), chondrogenic progenitor cells, chondrocytes (Zheng et al., 2019), PRP (), osteocytes	Reduce production of catabolic enzymes, promote chondrocytes to express cartilage ECM, (Guillén et al., 2021) promote chondrocyte differentiation (), promote proliferation of chondrocytes, inhibit chondrocyte apoptosis, (Lin et al., 2021)regulate immune response, or inhibit expression of inflammatory cytokines (Qiu et al., 2021)

Despite favorable safety profiles reported in early-phase studies, large-scale clinical translation of exosome therapy faces substantial barriers. Due to the lack of standardized surface markers, the co-isolation of other microvesicles during purification is inevitable, leading to variable purity across preparations, and no single ideal isolation method selective, cost-effective, high-yield, and reproducible currently exists (Jeyaraman et al., 2022). Manufacturing clinical-grade exosomes at scale is similarly constrained by the limited availability of GMP-certified laboratories for harvesting and production (Jeyaraman et al., 2022). Immunologically, exosomes derived from allogeneic or xenogeneic sources can elicit alloreactive T-cell responses, raising additional safety considerations for repeated dosing (Jeyaraman et al., 2022).

### Cell injection therapy

**FIGURE 5:**
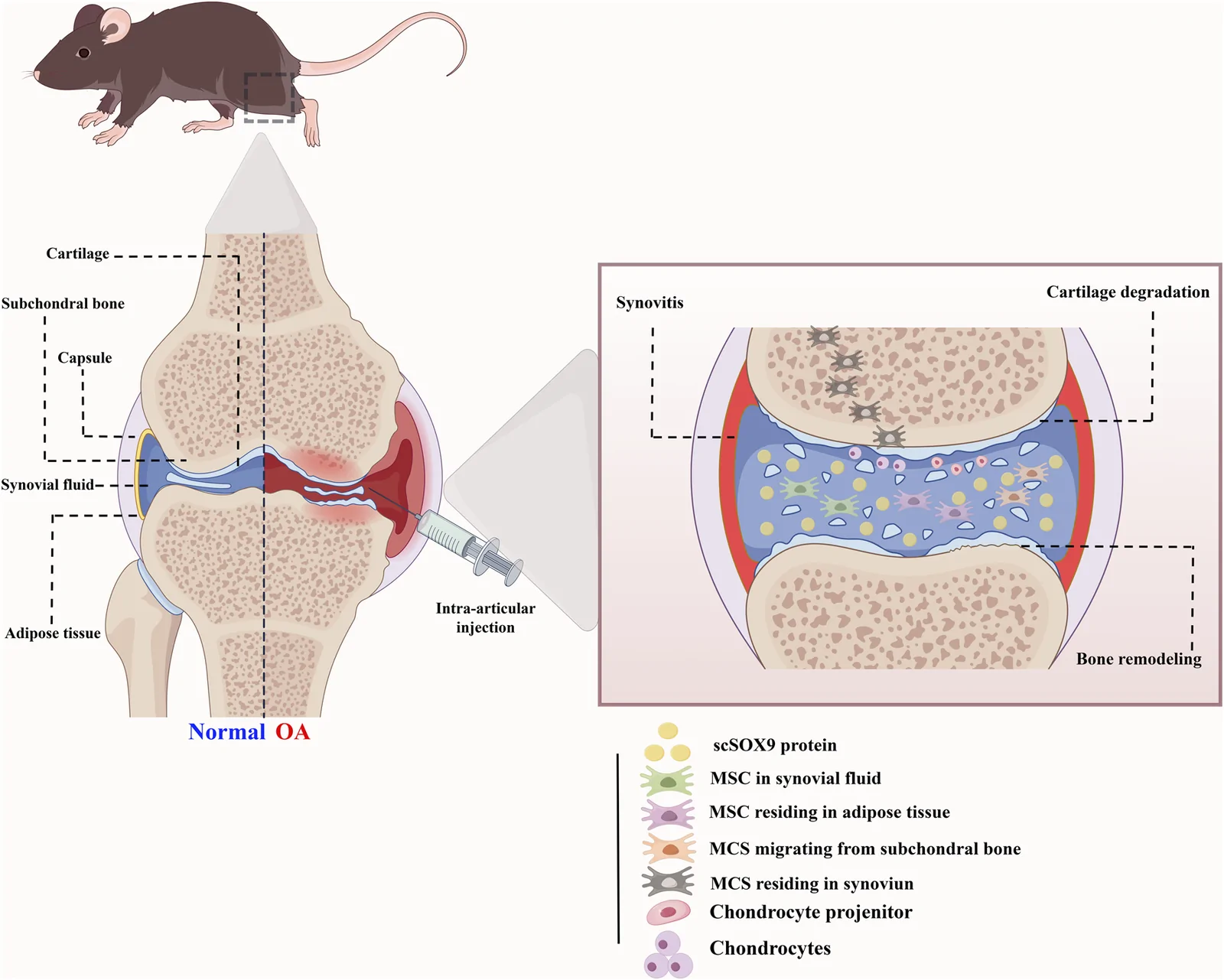
Overview of intra-articular MSC-based therapy in osteoarthritis, illustrating rodent OA models, synovitis, cartilage degradation, bone remodeling, and the disstribution of MSCs derived from synovial fluid, adipose tissue, synovium, and subchondral bone following injection.

Cellular therapies for OA increasingly favor MSCs over chondrocytes due to the procedural complexity and donor-site morbidity associated with chondrocyte-based approaches. As mesoderm-derived precursors capable of differentiating into adipocytes, chondrocytes, and osteocytes, MSCs can be isolated from multiple tissues, including adipose tissue, bone marrow, placenta, and peripheral blood, and offer superior chondrogenic potential, proliferation, and immunomodulatory activity. Their anti-inflammatory and immunosuppressive properties further support their ability to reduce synovial inflammation, alleviate pain, and function effectively as autografts or allografts in OA clinical trials (Im, 2018). MSC-based therapies demonstrate promising cartilage-regenerative effects across preclinical OA models, with intra-articular MSC delivery increasing cartilage thickness and structural repair in small-animal studies, while large-animal models such as sheep, horses, and goats provide more clinically relevant data crucial for evaluating translational efficacy (Sasaki et al., 2019) (Figure 5). *In vivo* studies demonstrate that IA delivery of autologous BM-MSCs can slow cartilage degradation in OA models, and genetically enhanced AD-MSCs expressing SOX-6 and SOX-9 further mitigate disease progression in goats. Clinical evidence supports the safety and feasibility of IA MSC therapy, with patients who have mild to moderate OA (Kellgren-Lawrence grades 1–3) appearing to be the most suitable candidates for these regenerative treatments (Ko et al., 2019). In a clinical trial involving 30 patients with chronic KOA, intra-articular injection of 40 × 10^6^ allogeneic BM-MSCs resulted in significant improvements in pain, function, and cartilage quality over 12 months compared with the control group treated with hyaluronic acid (Vega et al., 2015). However, MSC therapy appears less effective in patients with extensive cartilage damage, and MSCs harvested from individuals with advanced OA exhibit diminished proliferation and chondrogenic potential, producing fewer cells and showing a tendency toward osteogenic rather than cartilage-forming differentiation (Im, 2018).

### Comparative clinical maturity of regenerative modalities

The therapeutic strategies discussed above differ substantially in clinical maturity, regulatory status, and strength of supporting evidence (Table 3). PRP and BMAC represent the most clinically established modalities, supported by numerous completed randomized controlled trials, although preparation heterogeneity and modest effect sizes relative to one another remain unresolved (Glinkowski et al., 2025; Pintore et al., 2023). MSC-based therapies have advanced furthest among cell-based approaches, with a recent four-arm, phase 2/3 randomized trial directly comparing bone marrow aspirate concentrate, adipose-derived stromal vascular fraction, and allogeneic umbilical cord-derived MSCs against corticosteroid injection in 480 patients; at 1 year, none of the orthobiologic arms proved superior to corticosteroid injection or to one another, underscoring the gap between mechanistic promise and demonstrated comparative efficacy (Mautner et al., 2023). Exosome-based therapy remains the least clinically mature strategy, with evidence currently limited to first-in-human pilot dose-escalation studies (Wang et al., 2025). Gene therapy, by contrast, has progressed furthest toward potential regulatory approval among disease-modifying candidates: TissueGene-C, an allogeneic chondrocyte-based gene therapy overexpressing TGF-β1, has completed phase 2/3 evaluation and is among the most clinically advanced disease-modifying osteoarthritis drug (DMOAD) candidates identified in recent systematic reviews of the field, though no DMOAD has yet achieved regulatory approval (Brandt et al., 2025).

**TABLE 3:** Comparative clinical maturity, evidence, and regulatory status of OA regenerative modalities.

Modality	Clinical stage	Key evidence	Regulatory status	Primary limitation
PRP (Glinkowski et al., 2025)	Established clinical use; numerous completed RCTs	Multiple RCTs vs. HA show pain/function benefit, though heterogeneous results	Device (510(k)) for preparation kits; product itself not FDA-approved as a drug — used off-label	Heterogeneous preparation protocols; inconsistent efficacy across trials
BMAC (Pintore et al., 2023)	Established clinical use; comparative trials vs. PRP/ADSCs completed	Meta-analysis of 27 Level I studies: BMAC/PRP > HA; BMAC not superior to PRP	Autologous, minimally manipulated tissue — generally exempt from FDA drug pathway	Heterogeneous cell yield; no significant advantage over PRP demonstrated
MSCs (Mautner et al., 2023)	Phase 2/3 RCT completed; most advanced cell-based therapy	480-patient RCT: no orthobiologic arm (BMAC, SVF, UC-MSC) superior to corticosteroid injection at 1 year	No FDA-approved MSC product for OA	True hyaline cartilage regeneration not demonstrated; effect largely paracrine
Exosomes (Wang et al., 2025)	Earliest clinical stage; first-in-human pilot trial only	Single ascending-dose pilot trial (n = 41) shows safety, preliminary efficacy signal	No FDA-approved exosome product for any indication; classified as biologic requiring IND	No standardized isolation/manufacturing; large controlled trials lacking
Gene therapy (Brandt et al., 2025)	Most clinically advanced DMOAD candidate; phase 2/3 completed	TissueGene-C (TGF-β1-overexpressing chondrocytes) improved pain/function in phase 2/3 trials	No DMOAD yet approved by FDA; furthest along among DMOAD candidates	Long development timeline; allogeneic cell-gene manufacturing complexity

### Limitations and future directions

Although biologic and regenerative therapies have demonstrated symptomatic and mechanistic benefits in osteoarthritis, the barriers to their clinical translation vary substantially among therapeutic modalities and therefore require modality-specific optimization.

#### PRP and BMAC

The interpretation and comparison of PRP and BMAC studies remain limited by substantial heterogeneity in donor characteristics, preparation devices, platelet and leukocyte concentrations, cellular composition, activation procedures, injection frequency, and concomitant treatments (Murray et al., 2017; Magalon et al., 2016; Di Martino et al., 2022). Future investigations should adopt standardized reporting frameworks that quantify platelet, leukocyte, MSC, and growth-factor contents and should directly compare well-characterized formulations (Murray et al., 2017; Magalon et al., 2016; Di Martino et al., 2022). Stratified randomized trials are also required to determine whether therapeutic responses differ according to OA severity, inflammatory phenotype, age, metabolic status, and previous joint interventions. Single- and repeated-injection protocols should be compared using standardized patient-reported outcomes and structural imaging endpoints.

#### MSC-based therapies

Despite their immunomodulatory properties, allogeneic MSCs should not be regarded as completely immune-privileged. Experimental evidence indicates that allogeneic MSCs can induce donor-specific cellular and humoral immune responses, particularly after repeated administration, potentially accelerating cell clearance and reducing therapeutic persistence (Lohan et al., 2017; Barrachina et al., 2020). Future studies should therefore incorporate donor-recipient cross-matching, anti-donor antibody assessment, immunophenotyping, and comparative evaluation of autologous and allogeneic cell sources. Additional concerns include phenotypic instability during prolonged culture, cellular senescence, chromosomal abnormalities, and inappropriate differentiation. Although clinically significant ectopic ossification following intra-articular MSC administration has not been consistently demonstrated, the intrinsic osteogenic potential of MSCs and the possibility of hypertrophic or mineralized tissue formation warrant long-term surveillance. Manufacturing protocols should include passage limits, karyotypic and genomic stability testing, lineage-specific potency assays, and assessment of hypertrophic and osteogenic markers before administration. Large-animal studies and clinical trials should further evaluate cell persistence, biodistribution, dose-dependent effects, and the development of ectopic or calcified tissue using longitudinal imaging and histological analysis.

#### Exosome-based therapies

The clinical translation of exosome-based therapies is constrained by difficulties in producing reproducible, GMP-grade preparations at a clinically relevant scale. Exosome yield and composition are strongly affected by donor-cell source, passage number, culture medium, oxygen tension, bioreactor conditions, purification procedures, and storage. Conventional isolation methods may also co-isolate proteins, lipoproteins, protein aggregates, and other extracellular-vesicle populations, thereby producing preparations with inconsistent purity and biological activity. The MISEV2023 recommendations emphasize the need to characterize extracellular vesicles using complementary measurements of particle abundance, morphology, vesicle-associated proteins, and potential non-vesicular contaminants rather than relying on a single marker or isolation method (Welsh et al., 2024). Future studies should establish GMP-compatible manufacturing pipelines using xeno-free culture conditions, closed bioreactor systems, tangential-flow filtration, size-exclusion chromatography, or combinations of scalable purification techniques. Hollow-fibre and stirred-tank bioreactor systems have demonstrated the feasibility of increasing extracellular-vesicle yield while maintaining reproducible particle characteristics, supporting their further evaluation for clinical manufacturing (Goblin et al., 2021; Ulpiano et al., 2025). Standardized release criteria should include particle concentration and size distribution, morphology, positive vesicle-associated markers, negative contaminant markers, sterility, endotoxin and *mycoplasma* testing, cargo consistency, residual host-cell components, and validated functional potency assays. Dose-escalation studies should additionally evaluate repeated administration, immunogenicity, pharmacokinetics, intra-articular persistence, storage stability, and long-term safety.

#### Gene-based therapies

Viral-vector-mediated gene therapy presents several vector-specific safety challenges. Pre-existing or treatment-induced antibodies against viral capsids may neutralize vectors, reduce transduction efficiency, restrict repeated administration, or provoke innate and adaptive immune responses. These effects are particularly relevant to adeno-associated viral vectors, despite their comparatively favorable safety profile (Arjomandnejad et al., 2023; Yang et al., 2022). Unintended transduction of non-target tissues, prolonged or inadequately regulated transgene expression, insertional events, and off-target genome editing represent further concerns. These risks require particular consideration in OA because it is a chronic but generally nonfatal disease, making a highly favorable long-term benefit–risk profile essential. Future research should prioritize cartilage- or synovium-selective vectors, engineered capsids with improved joint tropism, tissue-specific or inducible promoters, and lower effective vector doses. Nonviral nanoparticles, messenger RNA delivery, and other transient gene-regulation approaches may be preferable when permanent or prolonged expression is unnecessary. For genome-editing strategies, comprehensive assessment should include unbiased off-target sequencing, insertion and deletion analysis, chromosomal rearrangement testing, biodistribution, vector shedding, germline-exposure assessment, and long-term tumorigenicity monitoring. Clinical protocols should additionally screen patients for pre-existing vector immunity and incorporate vector-specific immune monitoring, predefined stopping criteria, and extended post-treatment surveillance.

#### Biomaterial-supported delivery

The effectiveness of intra-articularly injected MSCs and exosomes may be limited by rapid dispersion through synovial fluid, lymphatic clearance, insufficient adherence to cartilage, and short residence within the joint. Biomaterial carriers, including injectable hydrogels, microgels, microspheres, fibrin-based matrices, and cartilage-adhesive systems-may protect therapeutic cargo, prolong intra-articular retention, and enable sustained or stimulus-responsive release. For example, an injectable hyaluronic acid/polyethylene glycol hydrogel was shown to provide controlled release of MSC-derived small extracellular vesicles while preserving their biological activity and improving therapeutic effects in experimental OA (Yang et al., 2021b). Similarly, thermosensitive hydrogels containing chondrocyte-derived exosomes promoted sustained exosome release, macrophage polarization, and cartilage repair in preclinical models (Sang et al., 2022). However, carrier selection should be matched to the biological properties of the therapeutic cargo. Cell-delivery systems should preserve viability, nutrient diffusion, cell–matrix interactions, and chondrogenic function while protecting cells from injection-associated shear stress. Exosome carriers should maintain vesicle integrity and biological activity while providing predictable binding and release kinetics. Biomaterials should also be evaluated for injectability, gelation time, cartilage adhesion, porosity, mechanical compatibility, degradation products, immunogenicity, and responsiveness to the inflammatory and enzymatic environment of the osteoarthritic joint. Future experiments should directly compare free and carrier-associated treatments using quantitative retention, biodistribution, release, toxicity, and efficacy assays. Promising systems should subsequently be evaluated in mechanically loaded large-animal joints before clinical translation.

## Conclusion

Biologic and regenerative therapies are redefining the management of articular cartilage degeneration, offering mechanistic depth beyond symptomatic care. While modalities such as MSCs, exosomes, PRP, and BMAC show therapeutic promise, their clinical utility is limited by variability in protocols and insufficient long-term data. Future efforts must focus on standardization, mechanistic validation, and controlled trials to enable safe, targeted, and durable integration into clinical practice.
